# How can we optimise inhaled beta2 agonist dose as ‘reliever’ medicine for wheezy pre-school children? Study protocol for a randomised controlled trial

**DOI:** 10.1186/s13063-016-1437-7

**Published:** 2016-11-11

**Authors:** Somnath Mukhopadhyay, Paul Seddon, Gemma Earl, Emma Wileman, Liz Symes, Cathy Olden, Corinne Alberti, Stephen Bremner, Alison Lansley, Colin N. A. Palmer, Nicole Beydon

**Affiliations:** 1 Academic Department of Paediatrics, Royal Alexandra Children’s Hospital, Eastern Road, Brighton, East Sussex BN2 3BE UK; 2 Brighton and Sussex Clinical Trials Unit, 16 Bloomsbury House, Bloomsbury Street, Brighton, East Sussex BN2 1HQ UK; 3 Haydn’s Wish Charity for Asthma and Allergy Research, 27 Valley Dene, Newhaven, East Sussex BN9 9NF UK; 4 AP-HP, Hôpital d’Enfants Robert Debré, Unité d’Epidémiologie Clinique and Inserm, CIE5, Paris, France; 5 Department of Pharmacy and Biomolecular Sciences, University of Brighton, Moulsecoomb, Brighton, East Sussex BN2 4GJ UK; 6 Pat Macpherson Centre for Pharmacogenetics and Pharmacogenomics, Medical Research Institute, Ninewells Hospital and Medical School, Dundee, DD1 9SY UK; 7 Unité Fonctionnelle de Physiologie-Explorations Fonctionnelles Respiratoires (EFR), Hôpital Armand-Trousseau, 26 Avenue du Docteur Arnold Netter, 75571 Paris Cedex 12, France

**Keywords:** Asthma, Wheeze, Children, Paediatric, Salbutamol, Dose finding

## Abstract

**Background:**

Asthma is a common problem in children and, if inadequately controlled, may seriously diminish their quality of life. Inhaled short-acting beta2 agonists such as salbutamol are usually prescribed as ‘reliever’ medication to help control day-to-day symptoms such as wheeze. As with many medications currently prescribed for younger children (defined as those aged 2 years 6 months to 6 years 11 months), there has been no pre-licensing age-specific pharmacological testing; consequently, the doses currently prescribed (200–1000 μg) may be ineffective or likely to induce unnecessary side effects. We plan to use the interrupter technique to measure airway resistance in this age group, allowing us for the first time to correlate inhaled salbutamol dose with changes in clinical response. We will measure urinary salbutamol levels 30 min after dosing as an estimate of salbutamol doses in the lungs, and also look for genetic polymorphisms linked to poor responses to inhaled salbutamol.

**Methods:**

This is a phase IV, randomised, controlled, observer-blinded, single-centre trial with four parallel groups (based on a sparse sampling approach) and a primary endpoint of the immediate bronchodilator response to salbutamol so that we can determine the most appropriate dose for an individual younger child. Simple randomisation will be used with a 1:1:1:1 allocation.

**Discussion:**

The proposed research will exploit simple, non-invasive and inexpensive tests that can mostly be performed in an outpatient setting in order to help develop the evidence for the correct dose of salbutamol in younger children with recurrent wheeze who have been prescribed salbutamol by their doctor.

**Trial registration:**

EudraCT2014-001978-33, ISRCTN15513131. Registered on 8 April 2015.

## Background

Asthma is a common problem in childhood that, if inadequately controlled, seriously diminishes quality of life [1]. Approximately one million UK children have been diagnosed with asthma, and almost all of them will have been prescribed a short-acting inhaled beta2 agonist (usually salbutamol) as a ‘reliever’ medication to help control their day-to-day symptoms. Inhaled salbutamol is thus one of the most commonly prescribed medicines for children in the UK and many other countries around the world.

The dose-response relationship of any medicine must be determined prior to regulatory approval. Such research is relatively straightforward to perform in adults, but more difficult for children. As a consequence, many medicines currently prescribed for children have not undergone age-appropriate testing and so may not be effective or may even induce unnecessary side effects. EU lawmakers have voted for this to change through an emphasis on such evaluation in children [2]. The bronchodilator efficacy of inhaled salbutamol in asthma has now been established in both older children and adults [3], as has the minimum effective dose in these two groups [4]. However, neither efficacy nor minimum effective dose has been established in younger children (defined as aged between 2 years 6 months and 6 years 11 months), despite inhaled salbutamol frequently being prescribed within this age group.

There are a number of consequences of our lack of knowledge in this area:

1. There is much uncertainty about the optimal dose of a beta agonist to use for symptom relief (for example, a variation between individual doses of 2–10 puffs is common practice).
2. There is uncertainty about the optimal dose of a beta agonist for measuring bronchodilator response in the diagnosis of asthma.
3. There is uncertainty about whether to use a beta agonist or alternatives such as ipratropium bromide or montelukast for symptom relief.

A dose of 2–10 puffs of inhaled salbutamol (approximately 100 μg per puff) is currently prescribed in both primary and secondary care as ‘reliever’ medicine for young children suffering from recurrent wheeze, a common feature of asthma in this younger age range. This dose is almost always administered via a spacer device, as younger children are unable to coordinate their breathing in order to inhale the medication directly from a metered dose inhaler. Following inhalation, the dose of salbutamol biologically available in the airways depends on many factors, such as breathing technique and airway calibre.

The urinary salbutamol level 30 min after the administration of an inhaled dose is a reasonable surrogate measure of the biologically available dose [5]. However, there are no studies where a specific effort has been made to collect a urine sample from a young child in the same manner.

The biologically available dose of salbutamol in the airway elicits the airway bronchodilator response. It is difficult to monitor side effects in young children, who are often unable to report discomfort such as palpitations or irritability. ‘Hyper-activity’ is a poorly studied problem that is commonly reported by parents of young children prescribed relatively large doses of salbutamol. In adults and older children, the response of the airway to a inhaled bronchodilator can be measured as changes in airway resistance or expiratory airflow before and after the administration of the bronchodilator. Most measures of airway resistance or airflow require the active co-operation of the patient, but such co-operation is difficult to achieve in young children. Conventional pulmonary function measurements, such as forced expiratory volume in 1 second or peak expiratory flow rate, either are impractical or are relatively unreliable in younger children. However, a technique relying on the transient interruption of breathing (known as the interrupter technique) has been recently developed as a reliable test of airway resistance (Rint) in this age group. Recent consensus guidelines [6] support the use of Rint in this age group for the measurement of airway resistance and for determining response to bronchodilator medication.

Thus there is now the possibility of correlating inhaled salbutamol dose with clinical response (measured as change in Rint) in younger children in the age range 2 years 6 months to 6 years 11 months. It is therefore of importance to document the airway response to increasing doses of inhaled salbutamol in this age group. To do this we need to document airway response against 30-min urinary salbutamol, the best available estimate for the bioavailability of salbutamol in the lungs. With expert children’s nurse support, we believe that most young children can be persuaded to pass a sample of urine at about 30 min following inhalation. However, we cannot assume that this will happen in all children and have made this a secondary outcome of the trial.

The proposed work is likely to identify a population of children who do not show a satisfactory response to salbutamol. There is evidence from studies in older children and young adults that one reason for this poor response may be a genetic change in their beta2 receptor (Arg/Gly16) [7, 8]. The presence of this polymorphism can be identified by DNA analysis on a simple saliva sample, without the need for specialist pulmonary function equipment. As these samples can easily be obtained in primary care, we propose to investigate whether this polymorphism is an effective marker for poor salbutamol efficacy in these younger children.

Overall, the proposed research will exploit simple, non-invasive and inexpensive tests that can mostly be performed in an outpatient setting in order to help develop the evidence for the correct dose of salbutamol in younger children with recurrent wheeze who have been prescribed salbutamol by their doctor. It is possible that a small dose of salbutamol, such as 2 puffs (200 μg) is adequate for most of these children. If this is the case, larger doses, such as 1000 μg, should not be prescribed (as is current practice) as they may induce side effects in these children. Secondly, some younger children who are currently prescribed larger doses of salbutamol may in fact be ‘poor responders’ or ‘non-responders’ to salbutamol due to their genetic constitution. Such children may benefit from alternative ‘reliever’ medicines, such as ipratropium or montelukast.

## Trial design

**Fig. 1:**
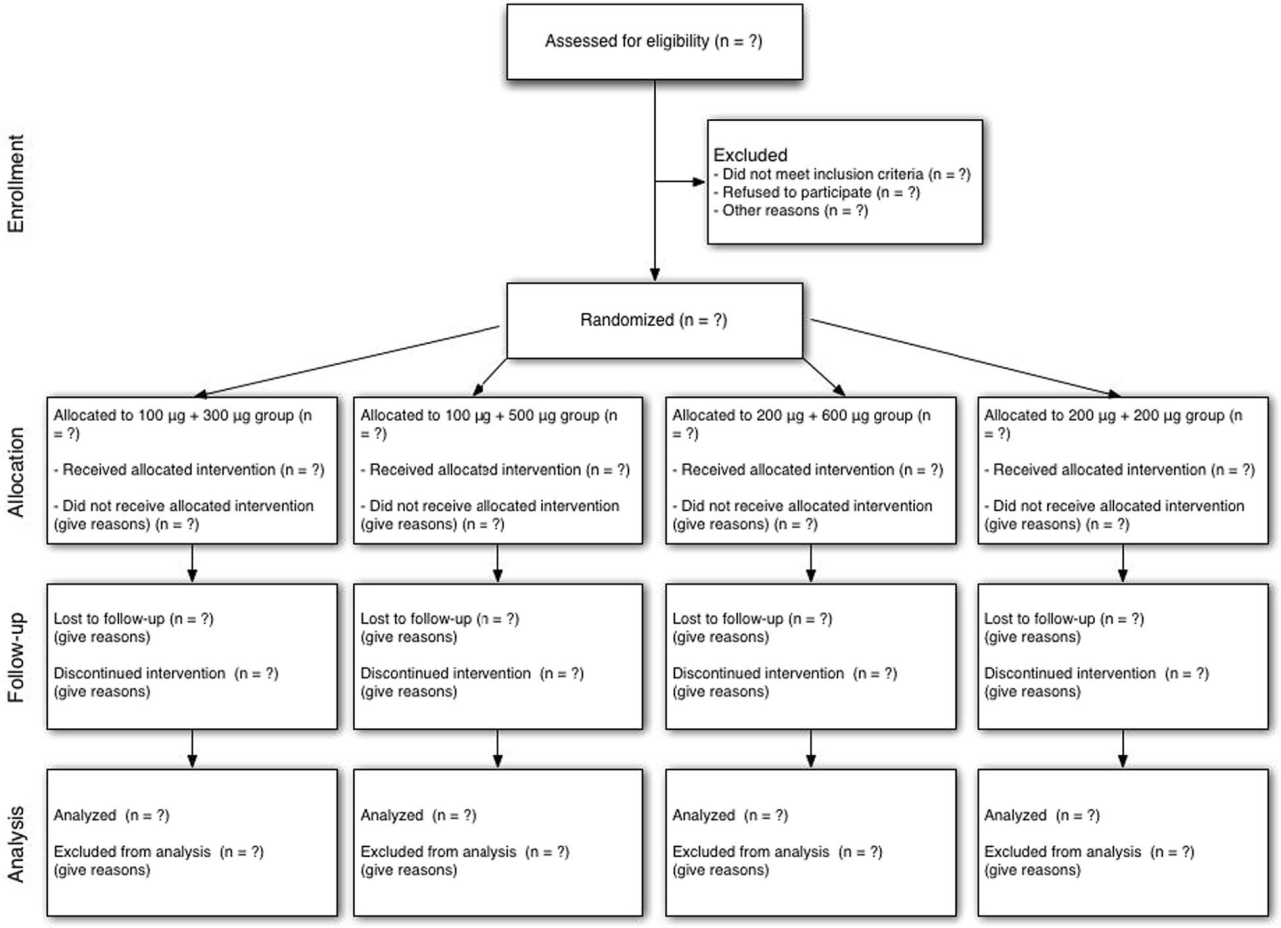
CONSORT flowchart for the OpSal trial

This is a phase IV, randomised, controlled, observer-blinded, single-centre trial with four parallel groups and a primary endpoint of the immediate bronchodilator response to salbutamol so that we can determine the most appropriate dose for an individual younger child. Simple randomisation will be used, with a 1:1:1:1 allocation (see the CONSORT flowchart of Fig. 1).

## Methods

### Participants, interventions and outcomes

Potential participants will be identified by members of the direct care team at the Royal Alexandra Children’s Hospital, Brighton in outpatient or inpatient wards or the children’s emergency department. The direct clinical care (doctor or nurse) team will then refer the patient and carer to the research nurse for further discussion regarding the study. If they are interested and are happy to meet the research nurse, this will be arranged. Where appropriate, the patient and carer are provided with a Patient Information Sheet (PIS). In some cases this material will be sent by post. An appointment is arranged at the hospital, where any further queries are addressed. Eligibility criteria are established by the doctor and consent obtained at this visit. Informed consent, where offered, will be received by the research nurse on the day the child attends the study. The research nurse will have already provided written information on the study to both carers and children so they will have had time to consider the information prior to attending their appointment with the research nurse. The nurse will also show pictures of the Rint and saliva collection equipment and demonstrate the equipment. We will ensure that the child and the carer fully understand the implications of the consent and that they understand that the consent is voluntary and will not affect their treatment rights.

#### Eligibility criteria

Inclusion criteria

The inclusion criteria are as follows:

- Children between the ages of 2 years 6 months and 6 years 11 months
- History of recurrent wheeze, defined as at least three episodes of wheeze over the previous 12 months

Exclusion criteria

The exclusion criteria are as follows:

- Other major airway or lung disease, e.g. physician-diagnosed chronic lung disease of prematurity, cystic fibrosis or abnormal airway anatomy
- Use of long acting beta2 agonists within 1 week of the study visit
- Treatment with systemic corticosteroids or leukotriene inhibitors within 2 weeks prior to the study visit.
- Involvement in a drug study within the last 30 days

### Measuring dose response to inhaled salbutamol using Rint

To span the entire plausible dose-response range in each individual child would be unethical, requiring an impractical long protocol and multiple Rint measurements, at an age when cooperation is notoriously difficult to achieve and maintain. We therefore plan to map out the dose-response curve using a sparse-sampling dosage schedule in which each individual child will receive just two successive doses, with Rint measurements at baseline and after the first and second dose. For the dose-response assessment using Rint, we used the same design as the one created by two of the investigators for a recent study (DORESI study NCT 01470755). There is an assumption that the measurements made after administration of the second dose reflect the sum of the first and second doses, i.e. that the two doses are additive, with no loss of potency for the first dose during the interval between the first and second measurements. The two doses to be administered in each individual child will be randomly determined from the options below and will be concealed from the researchers measuring the response using Rint. Children will be randomised to the four dosage schedules in the ratio 1:1:1:1.

### Dosage schedules

The dosage schedules are as follows:

1. 100 μg salbutamol → +300 μg = 400 μg salbutamol
2. 100 μg salbutamol → +500 μg = 600 μg salbutamol
3. 200 μg salbutamol → +600 μg = 800 μg salbutamol
4. 200 μg salbutamol → +200 μg = 400 μg salbutamol

Thus these four dosage schedules will contribute the following data points to the dose-response curve covering the range 100–800 μg (Table 1).

**Table 1:** Schedule of dose pairs to be tested

Dose studied	100 μg	200 μg	400 μg	600 μg	800 μg
1	√		√		
2	√			√	
3		√			√
4		√	√		

A schedule of assessments is given in Table 2.

**Table 2:** Schedule of assessments

Schedule items	Enrolment	Randomisation	Baseline	Testing: Dose 1	Testing: Dose 2
Time point				T_0_	T_45 min_
Enrolment					
Eligibility screening	X				
Informed consent/assent	X				
Allocation		X			
Interventions					
Dose 1				X	
Dose 2					X
Assessments					
Clinical history	X				
Recent relevant medications	X				
Adverse events				X	X
Saliva sample (for DNA work)			X		
Rint assessment			X	X	X
Urine sampling				X	X

Rint measurement will be performed at baseline and 15 min after each of the two doses of salbutamol. The second dose of salbutamol will be given 30 min after the first dose. If the participant needs to take a bronchodilator within 12 h of the study visit, then the research appointment should be rescheduled. Rint measurements will be made as specified in the consensus guidelines [6]. Briefly, the child will be seated and breathing quietly through the apparatus, via a mouthpiece, with nose occluded by noseclip or fingers and with cheeks supported. If the child is unable to tolerate or use a mouthpiece, a facemask will be used for all three measurements. At each Rint measurement, a minimum of 10 flow interruptions will be carried out, with inspection of mouth pressure transients in real time, in order to achieve a minimum of five technically acceptable interruptions. The Rint measurement will be calculated as the median value of all acceptable interruptions.

All participants will be prescribed a salbutamol inhaler from ring-fenced hospital stock, stored within the research office and dispensed by the research nurse. The inhaler will be labelled according to EU requirements. The salbutamol inhalers will not be temperature-monitored. An accountability log of the doses given, when and to whom will be kept by the research team. After administration of the protocol-defined dose, the inhaler will be returned to the research nurse, the accountability log updated and the inhaler transferred back to the clinical trial pharmacy for destruction. Salbutamol inhalers are licensed products, and for this trial they are being used in line with established practice, supported by guidelines, and the dosage schedule does not exceed what would be stated in the participant’s GP prescription.

### Collection of saliva for DNA analysis

Saliva samples will be collected in accordance with the instructions provided by the manufacturers of the sampling kits. Trial participants will be asked to spit directly into a container; for children unable or unwilling to spit we will collect saliva samples using swabs.

We will use the non-invasive Oragene® DNA collection kits (http://www.dnagenotek.com) for the collection of saliva for DNA extraction. The kit provides a median DNA yield of about 110 μg. The DNA from saliva is stable in this kit for up to five years at room temperature due to proprietary reagents that inactivate bacteria and nucleases in saliva and minimize chemical hydrolysis of DNA. The DNA will be stored in the Clinical Investigation and Research Unit, Brighton and Sussex University Hospitals Trust (BSUHT). It will be shipped as a single batch to the Medical Research Institute, University of Dundee at the end of the study for storage in their DNA bank prior to DNA extraction and Arg/Gly analysis at codon 16 of the beta2 adrenoreceptor gene.

### Collection of urine 30 min after each dose of inhaled salbutamol

We will endeavour to collect a urine sample from each trial participant 30 min after each dose of inhaled salbutamol, as an additional check on the exact dose delivered to the lungs and therefore absorbed into the circulation. Given the age of these children, it may be necessary to accept some variation in the timing of the sample. The research team includes an experienced paediatric nurse with expertise in gaining cooperation from very young children. The urine samples will be stored frozen at −80 °C in the Clinical Investigation and Research Unit (BSUHT) and at the end of the study shipped as a single batch to the School of Pharmacy and Biomedical Sciences (University of Brighton) for salbutamol analysis using liquid chromatography and mass spectrometry (LC/MS).

## Sample size

### Justification for research question 1: defining the optimal dose of salbutamol

Can we define the dose of inhaled salbutamol that is appropriate for children aged 2 years 6 months old to 6 years 11 months old with recurrent wheeze?

We plan to compare the difference in change from baseline between different salbutamol doses. From the data by Beydon et al. [9], we would define the minimum clinically important difference (MCID) for paired *t* tests as mean −0.23, standard deviation (SD) 0.19, and set the threshold of significance at 0.01 to account for multiple *t* tests. In order to detect a worthwhile difference of this size between dosages with 90 % power, a minimum of 24 patients will be needed in each dosage group, giving a sample size of 96 patients.

### Justification for research question 2: Arg versus Gly beta agonist response

Can we predict the salbutamol dose that is appropriate for an individual child using simple, non-invasive and inexpensive tests that can be performed in the outpatient setting?

The hypothesis to be tested is that children with wheeze who are either homozygous or heterozygous for arginine at position 16 of the beta2 receptor (i.e. Arg/Arg or Arg/Gly status) show a diminished peak response to inhaled salbutamol in comparison to children with wheeze carrying the Gly/Gly status.

The sample size calculations are based on data published by Beydon et al. [9]. Mean baseline Rint in asthmatic children was 0.92, SD 0.22 kPa/L/s; levels dropped to 0.74, SD 0.18 kPa/L/s after bronchodilator administration. In children who did not have asthma, the baseline Rint dropped by a smaller proportion, decreasing 0.10 unit to 0.82 kPa/L/s. All the children in this study will have wheeze. Hence, we feel they will have a higher baseline airway resistance comparable to children with asthma, and higher than controls without asthma, as reported in [9]. However, we estimate that children carrying one or both copies of Arg will show a diminished response that is comparable to that of children who do not have asthma. Thus our sample size calculations are based on the prediction that the peak bronchodilator response in the children who have Arg/Arg or Arg/Gly status will show a mean fall in Rint of 0.10 unit to 0.82 kPa/L/s, whereas the wheezy children who have Gly/Gly status will show a response that is at least as substantial as that observed by Beydon et al. [9] in children with asthma (i.e. a mean fall of 0.18 kPa/L/s).

The ratio of children carrying Arg/Arg and Arg/Gly status to those with Gly/Gly is 60 %:40 %. A sample size of 133 (80 Arg and 53 Gly/Gly) would allow us to detect a difference of 0.08 unit between the groups (delta = 0.08, SD = 0.20; effect size = 0.4) with 80% power for 5% significance, assuming a correlation between baseline and post-bronchodilator measurements of 0.6. To allow randomisation of equal numbers to the four groups, the sample size should be increased to 136. In order to allow a sample size sufficiently large to answer both research questions, and to allow for up to 12 % of children being unable to perform the measurement (based on experience in our unit in this age range), we will recruit a sample size of 156 children. We intend to recruit these children over 36 months.

### Study of the relationship between urinary salbutamol levels and Rint measurements

The concentration of salbutamol in the urine at 30 min following a dose of salbutamol represents the gold standard of lung bioavailability. This has been established in adults. We will try to collect as many urine samples as possible, and we will aim to study the dose that is available to the lungs, measured as the 30 min urine salbutamol, against bronchodilator response. Data analysis will be performed as discussed above for inhaled lung dose versus bronchodilator response. As the production of urine on request is rather uncertain in children of this age, this is intended as a distinct secondary analysis. The main project, including the principal outcomes, will not be affected in any manner if the collection of these samples is incomplete.

## Assignment of interventions

Participants will be randomised to the various pairs of salbutamol doses by a member of the research team using a web-based system (http://www.sealedenvelope.com/). Those delegated the role of randomising participants will be assigned a sealed.envelope.com randomisation user name and password by the trial manager. To randomise a participant, the randomiser will follow the OpSal Randomisation Guide. The randomisation system will be accessed at the following website: https://www.sealedenvelope.com/bsuh/users.

At the point of consent, a participant identifier will be assigned to the patient using the Participant Number Assignment Log. To randomise a participant, the participant identifier and date of birth must be entered, and then the inclusion and exclusion criteria must be confirmed. The participant is then randomised to a dosage schedule. An email notification is then sent to the randomiser and the coordinating centre.

An allocation sequence has been generated with four possible dosage schedules on a 1:1:1:1 basis. There is no stratification. The randomisation code list was generated by the trial statistician, and it was uploaded onto the web-based system by the trial manager. The direct research team members do not have access to the randomisation code list; therefore, concealment of allocation sequence is maintained until the moment of assignment.

The researcher performing the Rint measurement on the participant will be blinded to the dosing schedule until after the assessment has been performed. Two members of the research team will be required for the study visit. Researcher 1 will perform the Rint measurements and will be blinded to the dosage schedule. This researcher must leave the room whilst the salbutamol is being administered to the participant and any associated paper work must be concealed from this researcher until the study assessments have been performed. Researcher 2 will be unblinded to the dosage schedule. Researcher 2 will randomise the participant using the web-based randomisation service (sealedenvelope.com) on the day of the visit and will then administer the salbutamol. Once the final Rint measurement has been performed, the dosage schedule can be revealed to members of the research team if necessary.

In the unlikely event that the blinded researcher needs to know the dosage schedule given to the participant and unblinded members of the research team are not available to disclose it, the dosage schedule for each participant will be documented in the Case Report Form (CRF) and filed in the site file. The sponsor will also retain a copy of the randomisation list and will be notified of the dosage schedule at the point of randomisation via sealed.envelope.com.

## Data collection, management and analysis

### Data collection methods

The majority of data collected for the study will be recorded directly into the paper CRF. Source data entered directly into the CRF will include:

- Clinical history with a focus on wheeze and related atopic diseases
- Recent medication in relation to wheeze
- Study procedures: salbutamol dosing schedule, Rint measurements, saliva and urine sampling

The following information must be recorded in the participant’s health records in addition to the CRF:

- Eligibility
- Informed consent
- Trial number
- Adverse events and any other clinically significant information
- Withdrawal from the trial where appropriate

A copy of the original CRF should be kept in the participant’s health records. The CRF will be faxed or emailed to the Brighton and Sussex Clinical Trials Unit, Brighton, as per the study-specific CRF completion guidelines held in the site file. The original CRF will be stored in the site file.

Blank and completed copies of the paper CRF will be stored securely in the site file. Any deviations from the protocol will be noted on the CRF and documented in the Protocol Deviation Log stored in the site file.

The information collected during the study, including the genetic information, will be stored on a database. The information will be used solely for the purpose of research into childhood diseases related to inflammation. This may include sharing anonymised data from the study with researchers in France and Italy. Explicit consent from the parent will be obtained for this.

## Safety reporting (ICH Guideline E2A 1994)

An adverse event (AE) is defined as any unfavourable and unintended sign, symptom or disease temporally associated with the use of an investigational medicinal product or other protocol mandated intervention.

An adverse reaction (AR) is any untoward and unintended response to an investigational medicinal product related to any dose administered. All AEs judged by either the reporting investigator or the sponsor as having reasonable causal relationship to a medicinal product qualify as adverse reactions. The expression ‘reasonable causal relationship’ means to convey in general that there is evidence or argument to suggest a causal relationship (possible, probable or definite).

A serious adverse event (SAE) is any AE regardless of causality that:

- Results in death (during treatment with, and for 30 days after stopping, study drug).
- Is life-threatening, meaning that the patient was at immediate risk of death from the reaction as it occurred; i.e. it does not include a reaction which hypothetically might have caused death had it occurred in a more severe form.
- Requires hospitalisation or prolongs existing hospitalisation. Admissions and/or surgical operations scheduled to occur during the study period but planned prior to study entry or elective operations are not considered AEs if the illness or disease existed before the patient was enrolled in the trial, provided that it did not deteriorate in an unexpected manner during the trial (e.g. surgery performed earlier than planned).
- Results in persistent or significant disability or incapacity.
- Is a congenital anomaly/birth defect.
- Requires medical intervention to prevent permanent damage, or another medically important event, defined as an event that may not result in death, be life-threatening or require hospitalisation but may be considered an SAE when, based upon appropriate medical judgment, it may jeopardize the patient or subject and may require medical or surgical intervention to prevent one of the outcomes listed in the definitions for SAEs.

A serious adverse reaction (SAR) is defined as an SAE that has a definite, probable or possible causal relationship to the study drug (salbutamol). An adverse reaction is ‘unexpected’ if its nature and severity are not consistent with the information about the medicinal product set out in the Summary of Product Characteristics (SmPC). A suspected unexpected serious adverse reaction (SUSAR) is a drug-related SAR that has not previously been reported as a drug-related SAR (i.e. is not listed in the SmPC). Table 3 is taken from the salbutamol SmPC and lists the adverse events expected when taking salbutamol. This table should be referred to when determining the expectedness of an event. Frequencies are defined as: very common (≥1/10), common (≥1/100 and <1/10), uncommon (≥1/1000 and <1/100), rare (≥1/10,000 and <1/1000), very rare (<1/10,000) and not known (cannot be estimated from available data).

**Table 3:** Adverse events expected when taking salbutamol, taken from the SmPC

Adverse event	Common	Uncommon	Rare	Very rare
Immune system disorders		Hypersensitivity reactions (angio-oedema, urticaria, hypotension and collapse)		
Metabolism and nutrition disorders			Hypokalaemia	
Nervous system disorders		Headache	Hyperactivity, restlessness, dizziness	
Cardiac disorders	Palpitations			Myocardial ischaemia, cardiac arrhythmias including atrial fibrilation, supraventricular tachycardia and extrasystoles
Vascular disorders	Peripheral vasodilatation, and as a result small increase in heart rate			
Respiratory, thoracic and mediastinal disorders			Bronchospasm, cough, irritation of mouth and throat which may be prevented by rinsing the mouth after inhalation	
Musculoskeletal and connective tissue and bone disorders	Tremor		Muscle cramps	

All AEs will be documented in the hospital notes. ARs will be recorded in the hospital notes and in the CRF which will be filed in the participant’s hospital records. SAEs and SARs will be recorded in the hospital notes and the CRF and will be reported to the sponsor in an expedited fashion as per the relevant sponsor Standard Operating Procedure (SOP) for Safety Reporting. Whether expected or not, Table 4 illustrates how the events should be reported during the study. For each event the investigator or other medically qualified doctor on the delegation log will make a documented assessment of causality, seriousness and expectedness. If the research team becomes aware of any SARs once participation has stopped, these will be reported to the sponsor in an expedited fashion as per the relevant sponsor SOP for Safety Reporting.

**Table 4:** Procedure for reporting adverse events during the trial

Type of event^a^	Hospital notes	CRF	Expedited reporting (immediately and within 24 h)
Adverse event	Yes	No	No
Adverse reaction	Yes	Yes	No
Serious adverse event	Yes	Yes	Yes
Serious adverse reaction	Yes	Yes	Yes

^a^For definitions see section on Safety reporting (ICH Guideline E2A 1994) in main text

## Monitoring

### Data monitoring

A formal data monitoring committee will not be convened for this research study. There will be quarterly reviews of any adverse reactions and serious adverse events/reactions reported and appropriate action taken.

### Monitoring

The monitoring plan for the study will document the intensity of monitoring required.

### Auditing

The trial may be audited by the sponsor (BSUHT) in accordance with their annual audit plan.

## Statistical analysis plan

Rint measurements will be summarised, by dosage arm and by polymorphism, using means, standard deviations, medians and interquartile ranges. To answer research question 1, post-bronchodilator Rint will be regressed on baseline Rint to increase the precision of the treatment effect estimate, and dosage regimens with 100 + 300 mg of salbutamol taken as the reference group. Adjusted differences in mean Rint will be reported together with 99 % confidence intervals and *p* values, with *p* < 0.01 chosen to be significant in light of the multiple testing. To answer research question 2, extending the above model, a binary variable indicating homozygosity or heterozygosity for arginine and the interaction terms between this variable and the dosage regimen indicator variables will be included in the model.

## Ethics and dissemination

### Research ethics favourable opinion

A favourable opinion from a national research ethics committee (REC) has been received from the East of Scotland Research Ethics Service REC 2 (reference 14/ES/0072) prior to obtaining NHS permissions and commencing the study.

### Protocol amendments

Any proposed amendments to the research study will be discussed with the sponsor. The sponsor will determine whether the amendment is substantial or not. If the amendment is deemed substantial, the sponsor will determine whether the amendment needs review by the REC, the Medicines for Healthcare Regulatory Agency (MHRA) or both. The Principal Investigator is responsible for ensuring that the amendments are implemented and important modifications are communicated to relevant parties when applicable.

### Confidentiality

Each participant will be allocated a participant identifier number at the time of consent. Thereafter, this unique identifier will be used to identify the participant. The code list that details which participant identifier number links with which participant will be kept in a secure, swipe-access-only area in a locked room.

### Dissemination policy

The full trial protocol is being published in the online journal *Trials* (www.trialsjournal.com). A research summary will be provided to participants if they request to be made aware of this. Trial results will be communicated to healthcare professionals, the public and other relevant groups at conferences and press releases, through published papers in scientific journals and via parent groups. The primary trial publication will follow CONSORT reporting guidelines.

## Trial sponsor

The trial sponsor is (reference 150400) Brighton and Sussex University Hospitals NHS Trust (BSUHT), Royal Sussex County Hospital, Eastern Road, Brighton BN2 5BE, UK. The sponsor’s representative is Mr Scott Harfield, BSUHT Research and Development Manager, telephone + (44) 01273 696955, email scott.harfield@bsuh.nhs.uk.

The sponsor (BSUHT) has had no role in the design of this study. Their role in the study will be limited to governance issues such as determining whether any proposed amendments to the current protocol are substantial or not. Other than this governance role, the sponsor will not be involved in the execution of the study, data analyses, interpretation of the data or the decision to submit results for publication. The funder’s reviewers have made various suggestions in terms of study design but will not have any role during its execution, data analyses, interpretation of the data or the decision to submit results for publication.

## Committees

### Trial Steering Committee (TSC)

The TSC consists of Gemma Earl (Chair), Somnath Mukhopadhyay, Paul Seddon and Emma Wileman. The TSC will provide overall supervision for the trial, monitoring its progress and ensuring adherence to the protocol and the principles of Good Clinical Practice (GCP). The TSC will focus on recruitment, adherence to the protocol, participant safety and any considerations based on new information emerging during the trial.

### Data monitoring committee (DMC)

As this trial involves a licensed medicine with an extensively researched safety profile that will be previously have been used by all potential participants, the sponsor has agreed that a DMC will not be convened.

## Trial status

The trial is now open, with 37 children recruited as of June 2016.

## Abbreviations

Rint, airways resistance using interrupter technique
